# Perfecting practice: a protocol for assessing simulation-based mastery learning and deliberate practice versus self-guided practice for bougie-assisted cricothyroidotomy performance

**DOI:** 10.1186/s12909-019-1537-7

**Published:** 2019-04-05

**Authors:** Andrew Petrosoniak, Marissa Lu, Sara Gray, Christopher Hicks, Jonathan Sherbino, Melissa McGowan, Sandra Monteiro

**Affiliations:** 1 Department of Emergency Medicine, St. Michael’s Hospital, 30 Bond Street, Toronto, Ontario M5B 1W8 Canada; 2 Division of Emergency Medicine, Department of Medicine, University of Toronto, Toronto, Canada; 3 Faculty of Medicine, University of Toronto, Toronto, Canada; 4 Interdepartmental Division of Critical Care, University of Toronto, Toronto, Canada; 5 McMaster Education Research, Innovation and Theory (MERIT) program, McMaster University, Hamilton, Canada

**Keywords:** Medical education, Deliberate practice, Mastery learning, Simulation-based medical education, Self-guided practice, Cricothyroidotomy, Procedural skill

## Abstract

**Background:**

Simulation-based medical education (SBME) is a cornerstone for procedural skill training in residency education. Multiple studies have concluded that SBME is highly effective, superior to traditional clinical education, and translates to improved patient outcomes. Additionally it is widely accepted that mastery learning, which comprises deliberate practice, is essential for expert level performance for routine skills; however, given that highly structured practice is more time and resource-intensive, it is important to assess its value for the acquisition of rarely performed technical skills. The bougie-assisted cricothyroidotomy (BAC), a rarely performed, lifesaving procedure, is an ideal skill for evaluating the utility of highly structured practice as it is relevant across many acute care specialties and rare – making it unlikely for learners to have had significant previous training or clinical experience. The purpose of this study is to compare a modified mastery learning approach with deliberate practice versus self-guided practice on technical skill performance using a bougie-assisted cricothyroidotomy model.

**Methods:**

A multi-centre, randomized study will be conducted at four Canadian and one American residency programs with 160 residents assigned to either mastery learning and deliberate practice (ML + DP), or self-guided practice for BAC. Skill performance, using a global rating scale, will be assessed before, immediately after practice, and 6 months later. The two groups will be compared to assess whether the type of practice impacts performance and skill retention.

**Discussion:**

Mastery learning coupled with deliberate practice provides systematic and focused feedback during skill acquisition. However, it is resource-intensive and its efficacy is not fully defined. This multi-centre study will provide generalizable data about the utility of highly structured practice for technical skill acquisition of a rare, lifesaving procedure within postgraduate medical education. Study findings will guide educators in the selection of an optimal training strategy, addressing both short and long term performance.

## Background

There is substantial evidence that SBME is a superior training technique compared to traditional didactic methods for technical skill acquisition [1, 2]. As both technology and patient care become increasingly complex, simulation-based medical education (SBME) provides a feasible alternative allowing trainees to practice without harming patients [3]. Unfortunately, SBME is a resource intensive approach to medical training, requiring careful alignment of education and practice design principles with the intended outcomes. For example, there is growing evidence that deliberate practice and mastery learning approaches to training for procedural skills can ensure expert level performance, particularly for routine procedures [4–7]. Applying this structured approach, learners can transition as outlined in the “Dreyfus model” along a continuum of five stages: novice, advanced beginner, competent, proficient and expert [4]. Highly structured practice requires a substantial commitment of time and instructional resources [8]. Identifying the most effective and evidence-based SBME methods for rare procedures is a critical task for educators in acute care medicine [9, 10].

### Deliberate practice and mastery learning (DP + ML)

Deliberate practice (DP) is an instructional method widely regarded as the mainstay for expert skill acquisition [11–13]. First described by Ericsson and his colleagues, this method refers to engagement in structured activities with the goal to improve performance in a domain, through an iterative cycle of practice, feedback, and successive refinement [14]. It is based on 4 key components [11]:

1. Motivated learners
2. Well-defined goals for improvement
3. Ample opportunity to practice through repetition
4. During practice, focused feedback is provided with skill adjustments made accordingly

**Fig. 1:**
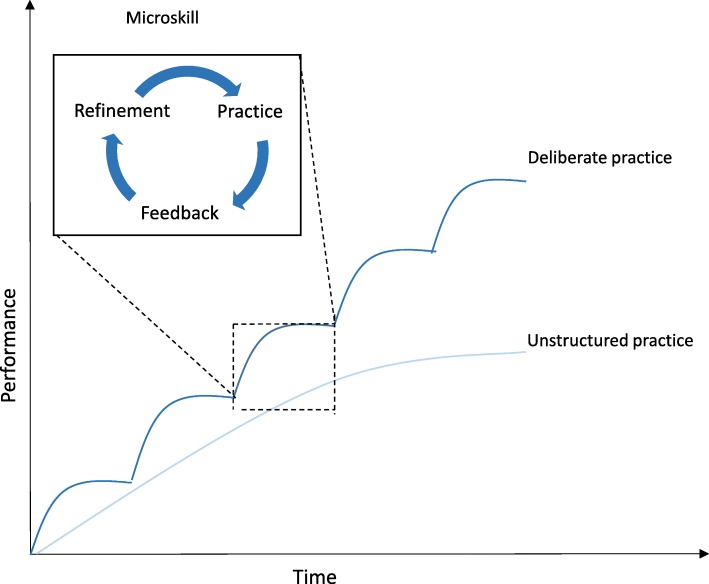
Deliberate practice model. Deliberate practice occurs over time with repeated feedback and refinement of microskills, combined together resulting in improved task completion

Deliberate practice is often coupled with the mastery learning (ML) model, where tasks are broken into a series of smaller and progressively more complex microskills [15]. Learners advance through each of the tasks by applying skills they have acquired during preceding steps [16] (Fig. 1). Deliberate practice and masterly learning (DP + ML) improves performance across a variety of disciplines including sports and music and there is growing evidence of its effectiveness within medical education [2, 12, 17].

Some authors suggest that simulation training using DP + ML results in superior outcomes compared to other methods of simulation-based practice [2]. An alternative to DP + ML is self-guided practice (also referred to as self-directed or self-regulated) whereby “*individuals take the initiative, with or without the help of others in diagnosing their learning needs, formulating learning goals, identifying human and material resources for learning, choosing and implementing appropriate learning strategies, and evaluating learning outcomes*” [18]. The few studies that compare DP with self-guided practice yielded mixed results while also limited by both small sample sizes and generalizability of a single institution setting [8, 19].

An important confounding factor that may explain these discrepant results is the variability in how DP is applied during SBME. Deliberate practice is often described in the context of sustained investment in practice over long periods of time in order to attain expert status [9]. In medical training, this can be difficult given the wide breadth of skills and knowledge that are required, especially when it comes to rarely performed procedures. While most clinical educators adhere to the key components of Ericsson’s criteria, in real practice, training is condensed over a shorter time-frame. The goal of the current study therefore is to explore the application of DP in a realistic context in order to understand how it is best optimized in curricular design, particularly for the acquisition of a rarely performed technical skill in the emergency medicine setting. Our study design incorporates several metrics of performance including a global rating scale and chronometry measured both immediately after and 6–12 months following skill instruction.

### Chronometry

Chronometry, the measurement of time, is an important metric for time sensitive procedures yet it remains uncommonly applied within technical skill training curricula [20]. However, introducing procedural time-based measurements can further improve deliberate practice by facilitating overlearning (going beyond the minimal level of competence required), increasing the challenge level, and providing additional feedback which can enhance a learner’s self-assessment and increase motivation [20].

### Retention

A key aspect of procedural skill acquisition is skill retention. Medical trainees are expected to demonstrate competence in performing a wide array of procedures, despite having limited exposure to practice opportunities [21]. Acute care physicians face the additional challenge of performing these procedures with little to no warning in stressful situations with unstable patients.

Several studies have looked at SBME to improve skill retention yielding variable results. Skill decay ranges from 2 weeks to 14 months for procedures such as ACLS, shoulder dystocia and cricothyroidotomy skills, with the most common retention interval being 6–12 months [22–25]. These differences are perhaps unsurprising, given that there are a number of different factors that influence retention including degree of overlearning, performance conditions during retention evaluation, task characteristics, and unmeasured individual differences [26–30]. While it appears that 6–12 months is a reasonable timeframe to expect for skill retention, there is uncertainty regarding the training approach best suited to optimize skill performance over time.

### Bougie-assisted cricothyroidotomy

The cricothyroidotomy is a life-saving procedure performed as a final option in all emergency airway algorithms [31, 32]. It represents an ideal prototype to evaluate the utility of DP + ML in skill acquisition and retention as it is rare, time-sensitive, and relevant across most acute care, hospital-based specialties [31, 33–36]. Furthermore, the recent introduction of a novel technique—the bougie-assisted cricothyroidotomy (BAC)—offers additional opportunity as residents are unlikely to have previous training or experience.

#### Justification/summary

Large scale, multi-centre, comparative effectiveness studies of SBME methods are needed to better define optimal simulation teaching strategies. This study will compare DP + ML with self-guided practice on the acquisition and retention of a rarely performed skill –bougie assisted cricothyroidotomy. To our knowledge, this is the first multi-centre, randomized study to evaluate the role of DP + ML on skill acquisition and retention.

## Methods

### Aim/objective

The objectives of this study are to:

- Compare two instructional techniques on the skill performance of a bougie-assisted cricothyroidotomy immediately and after 6–12 months after training
- Evaluate participant attitudes and preferences following two instructional techniques and practice

### Study design and setting

**Fig. 2:**
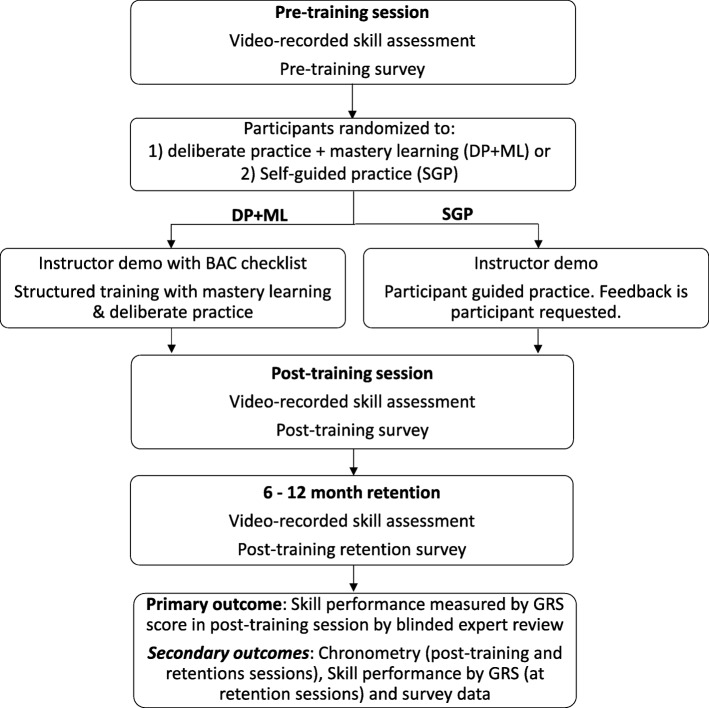
Study design

This is a multi-centre, blinded, randomized study (Fig. 2). The study will take place at five emergency medicine (EM) residency programs across North America (4 Canadian- and 1 US-based centres). All EM residents PGY1–5 are eligible and will be invited to participate. Written consent will be obtained from all study participants. There are no exclusion criteria. We received research ethics board approval from all participating institutions.

### Instructional methods

Residents at each study site will be assigned to receive either DP + ML, or self-guided practice using a computer-generated randomization process. Participants will be blinded to the study group assignments. For practical purposes, we will cluster 4–6 participants with 1 instructor who will facilitate the training using one of the two instructional methods. This group size aligns with recommendations from surgical education literature for procedural skill training that demonstrates the optimal trainee:instructor ratio for procedural skills training [37]. All instructors are board certified emergency physicians with expertise in procedural skill training. Prior to the session, each instructor will receive an introduction to the task trainer, a briefing on how to conduct the training and a standardized approach to BAC performance. The instructors assigned to the DP + ML session will also receive an introduction to the BAC checklist and explicit instructions to facilitate mastery learning followed by deliberate practice [38]. The instructors assigned to the self-guided practice session will be told explicitly that they can only provide feedback following a participant request. They will be instructed by the study investigators on the necessary steps for BAC performance.

Both groups will receive an introductory lecture to surgical airway performance and a demo of the BAC on the task trainer by the session instructor. Participants will then complete a pre-training survey assessing self-confidence and prior cricothyroidotomy experience, followed by a video-recorded performance of a BAC using an upper body task trainer (head and neck from a Laerdal SimMan) to assess baseline performance before training begins.

#### Deliberate practice and mastery learning

In the DP + ML group, the instructor will demonstrate the procedure following the list of essential steps, published previously [38]. These steps were selected using a modified Delphi methodology. Participants will perform each step under direct instructor observation in keeping with mastery learning techniques [8] and subsequent practice will follow principles of deliberate practice – with the goal of skill improvement using repetitive performance coupled with personalized feedback from the instructor. Chronometry will be introduced in later stages of practice through timed performance [20]. We chose to incorporate chronometry as an additional pedagogic intervention in later stages of learning to further incentivize practice with an objective feedback metric. The training session will be considered complete once the participant and instructor independently agree that competent skill performance is achieved.

#### Self-guided learning

In the self-guided learning group, participants will observe the procedure demonstrated by an instructor, followed by self-guided practice using the same equipment and working environment as the DP + ML group. During practice, feedback will only be provided following an explicit participant request. Participants will be informed that they can complete their training at their own discretion, once they report feeling comfortable with the procedure.

Finally, participants from both groups will perform a video-recorded attempt immediately following completion of their respective practice sessions and complete a post-training survey.

### Skill retention

Skill retention will be evaluated at 6 to 12 months following the initial training session. Due to logistical challenges related to resident schedules (e.g. away on elective, off-service), we require a 6 month time frame to maximize participation. Each participant will perform a video-recorded BAC on the same task trainer used during their training sessions followed by a post-training retention survey.

### Primary outcome

The primary outcome is the difference in skill performance of BAC between the two groups at post-training, assessed using a previously validated 7-point global rating scale (GRS) [22, 39]. Video recordings will be scored by three independent airway experts (2 Royal College certified emergency physicians, 1 staff anesthesiologist) trained in BAC. All reviewers will receive training from study investigators followed by practice video review to ensure scoring standardization. All reviewers will be blinded to the subjects’ identities and group allocation.

### Secondary outcomes

Secondary outcomes include participant feedback and chronometry after the initial training and retention sessions. Performance time begins when the participant first palpates the neck of the task trainer and ends with successful ventilation. Time scores will be measured using video review. Participant feedback will be collected with pre and post-training surveys. Skill performance (measured using GRS) at 6–12 months will also be compared.

### Sample size determination

A justification of sample size requires knowledge of results from similar studies. As there have been no studies of this exact design conducted previously we have used a sample size formula to evaluate our proposed sample size [40]. We base the sample size calculation on assumptions inherent of a between groups comparison [40]. Given the multi-site nature of this study, there will be approximately 16–20 participants in each site; for a total of 80 in each group. At 80 participants in each group, there is sufficient power (80%) to detect a difference in GRS score of 1.3 between groups, assuming α = 0.05 and β = 0.2 to detect. Similar approaches have been used by Freidman et al. [39] and Naik et al. [41].

### Statistical analysis

The survey data will be analyzed using descriptive statistics. The primary outcome (GRS) score will be evaluated using a repeated measures ANOVA with between subject factor of instruction (DP + ML or SG group) with one within subject factor of assessment repeated 3 times (pre, post, and retention). SPSS software (SPSS Inc. 18.0, Chicago IL) will be used for data analysis. 2-sided *P* < 0.05 is considered statistically significant for the primary outcome. Interrater reliability (IRR) for the reviewers’ scores will be analyzed using an intraclass correlation, after reviewing the same 20 videos. If a target IRR of ≥0.7 is achieved, the rest of the videos will be scored by one reviewer. Confidence ratings will be compared with GRS at pre-training, post-training and retention using Spearman’s correlations.

## Discussion

The unique challenges of postgraduate training in medicine demands that research seeks to enhance our understanding of optimal simulation-based instructional methods rather than further highlighting the benefits of hands-on practice when compared to non-simulation-based teaching [42]. While the benefits of ML + DP in simulation are promising, we should acknowledge that it is a complex intervention with a variety of elements that differ as a result of local circumstances and resource availability [2]. The resources required to implement DP + ML cannot be understated. There is often more equipment, trainers, and time required with this method [15]. In a similar study comparing simulation-based deliberate practice with self-guided practice on ultrasound regional anesthesia skills, subjects in the self-guided practice group spent an average of 6.8 min practicing compared to 48.2 min in the deliberate practice group.

Currently, there is a paucity of data to inform preferred methods of simulation-based procedural training. McGaghie et al. concluded in their 2011 meta-analysis that “only a small number of studies were identified that address head-to-head comparative effectiveness of SBME with deliberate practice and traditional clinical education or a preintervention baseline” [2]. A more recent systematic review and meta-analysis concluded that “limited evidence suggests” the superiority of mastery learning SBME over other techniques [15]. The authors noted that few studies compare ML with other simulation-based instructional methods, such as self-guided practice, with mixed results. In one study, anesthesia residents learning ultrasound guided regional anesthesia were randomized to either DP + ML or self-guided practice. The authors reported that while performance improved among both groups, there were no differences in participant skill performance or retention between groups [8]. In contrast, a study of medical students learning ultrasound guided regional anesthesia techniques concluded that the deliberate practice resulted in fewer errors compared to self-guided practice [19]. Both of these studies are subject to important limitations including a small sample size at a single institution. There remains uncertainty as to whether these findings can be extrapolated among a larger study population across multiple sites. Recently, a large, comparative, single site study of undergraduate medical trainees concluded mastery learning with simulation is superior to simulation without mastery learning for peripheral venous catheter insertion [43]. This certainly supports the use of ML in the undergraduate setting, however, its utility in postgraduate training requires further study.

Our study addresses this by comparing two different simulation-based instructional methods for EM residents using a large sample size at multiple centres. This study design seeks to overcome the limitation of generalizability—common among other single site SBME-based studies. In addition, the rarity and relative novelty of the BAC procedure itself minimizes baseline variation in prior experience skill.

This study has several limitations. Given that both teaching and testing occur with a low-fidelity simulator, our study is designed only to assess procedural skills rather than the decision making and non-technical skills of an interprofessional resuscitation. Due to logistical constraints, retention data collection will take place between 6 to 12 months rather than at a single point in time. Although all study participants will voluntarily enrol, we cannot guarantee their motivation to learn and improve, which is a key aspect of deliberate practice.

In conclusion, this study will have important implications for residency training as it will provide insight for medical educators involved in the design of simulation-based technical skills training. High quality simulation-based skills training is a promising method to improve clinical outcomes and patient safety [44]. At a system-based level, this study will aid administrators and medical education leaders in their decision making for resource allocation towards skills training and inform future curricular designs.

## Abbreviations

BAC: Bougie-assisted cricothyroidotomy
DP: Deliberate practice
GRS: Global rating scale
IRR: Interrater reliability
ML + DL: Mastery learning and deliberate practice
ML: Mastery learning
SBME: Simulation-based medical education
